# Stent Fracture and Perforation During Percutaneous Coronary Intervention for In-Stent Restenosis

**DOI:** 10.1016/j.jaccas.2025.104682

**Published:** 2025-08-20

**Authors:** Safi U. Khan, Hassaan B. Arshad, Neal S. Kleiman, Alpesh R. Shah

**Affiliations:** Section of Interventional Cardiology, Houston Methodist DeBakey Heart and Vascular Center, Houston Methodist Hospital, Houston, Texas, USA

**Keywords:** coronary perforation, in-stent restenosis, stent fracture

## Abstract

**Background:**

Stent fracture is a rare but potentially life-threatening complication of percutaneous coronary intervention (PCI). We present a case of type IV stent fracture during PCI of recurrent in-stent restenosis (ISR) of the right coronary artery.

**Case Summary:**

A 64-year-old man with prior coronary artery bypass grafting and recurrent ISR in the right coronary artery underwent PCI involving cutting balloon angioplasty and brachytherapy. Aggressive balloon dilation to achieve optimal results led to a type IV stent fracture with Ellis type III perforation. Prolonged balloon tamponade failed, and a covered stent was successfully deployed to seal the perforation.

**Discussion:**

This case highlights the risk of mechanical complications during the management of ISR and the importance of immediate intervention using covered stents in coronary perforations. We discussed case-relevant risk factors and management options for stent fracture and coronary perforation.

**Take-Home Messages:**

Operators must remain vigilant for rare complications like stent fractures and perforation in PCI of ISR.

## Graphical Abstract

**Figure:**
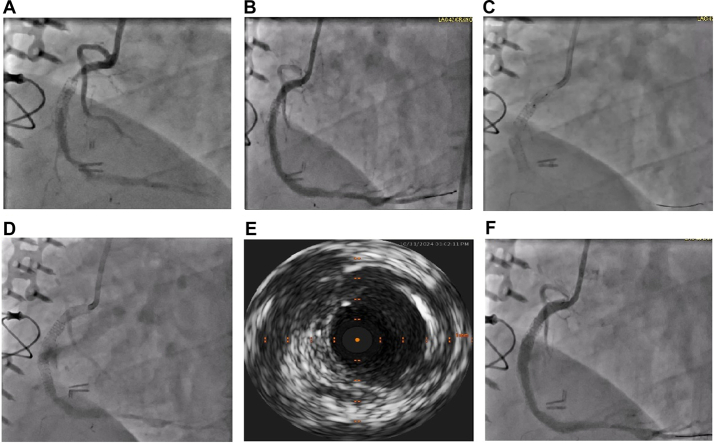

## History of Presentation

A 64-year-old man presented to the clinic for evaluation of recurrent exertional chest pain and shortness of breath. He had symptoms consistent with the Canadian Cardiovascular Society and NYHA functional class III, despite optimal medical therapy. Physical examination revealed no signs of heart failure or hemodynamic instability.

**Take-Home Messages**

• Type IV stent fractures are rare but can lead to life-threatening complications like coronary perforation.
• Risk factors include overlapping stents, aggressive postdilation, and adjunctive therapies such as brachytherapy.
• Covered stents are the preferred treatment for Ellis type III perforations when balloon tamponade fails.
• Operators should carefully match balloon diameter to stent architecture in previously treated segments.

## Past Medical History

The patient had a history of smoking, hypertension, hyperlipidemia, and type 2 diabetes mellitus. The patient had coronary artery disease status post coronary artery bypass graft surgery with prior angiograms showing patent right internal mammary artery (IMA) to the left anterior descending artery (LAD), patent left IMA to the ramus artery, moderate disease in left circumflex artery, and occluded saphenous vein graft to the right coronary artery (RCA). The patient had several prior percutaneous interventions to RCA.

## Differential Diagnosis

The clinical presentation raised the possibility of recurrent in-stent restenosis in RCA, graft failure, or progression of atherosclerotic disease in native coronary vessels. Noncardiac etiologies for chest pain were also considered in differential diagnosis, although with a lower degree of clinical probability.

## Investigations

**Figure 1:**
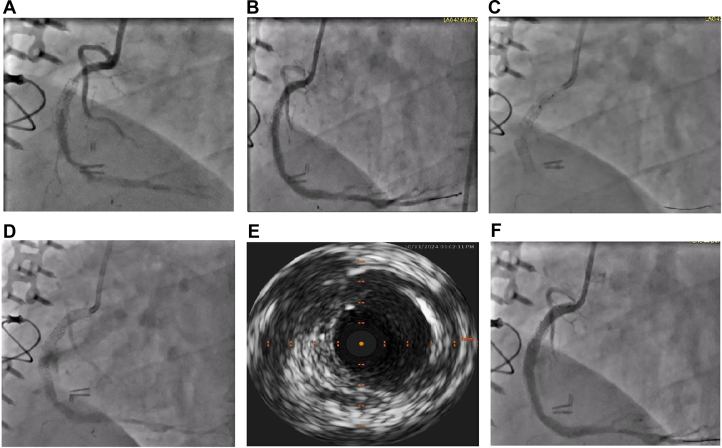
Coronary Angiogram of the Right Coronary Artery (A) Preintervention image; (B) postballoon dilatation and brachytherapy image; (C) stent IV fracture; (D) perforation; (E) intravascular ultrasound image showing perforation with stent gap; and (F) covered stent placement.

His electrocardiogram was unremarkable for ischemia. Baseline labs, including troponin levels, were within normal limits. His echocardiogram demonstrated normal left ventricular ejection fraction, grade I diastolic dysfunction, and no valvular lesions. Coronary and graft angiography revealed a moderate disease in mid LAD. The right IMA to LAD was patent with competitive flow in LAD; there was no significant disease distal to the anastomotic site of right IMA to LAD. LAD gave rise to 2 diagonal arteries without significant disease. Left circumflex artery was a nondominant vessel with 2 medium-sized obtuse marginal branches without significant disease. Ramus had a significant disease in the mid portion; left internal mammary artery to ramus was widely patent with competitive flow. RCA had 2 layers of stents in the proximal to mid portion and 1 stent in the distal portion. The proximal-to-mid stent had subtotal occlusion (Figure 1A). There was significant stenosis in the distal stent of RCA.

On hemodynamic assessment, left ventricular end-diastolic pressure was 20 mm Hg with no significant gradient across the transaortic valve.

## Management

**Figure 2:**
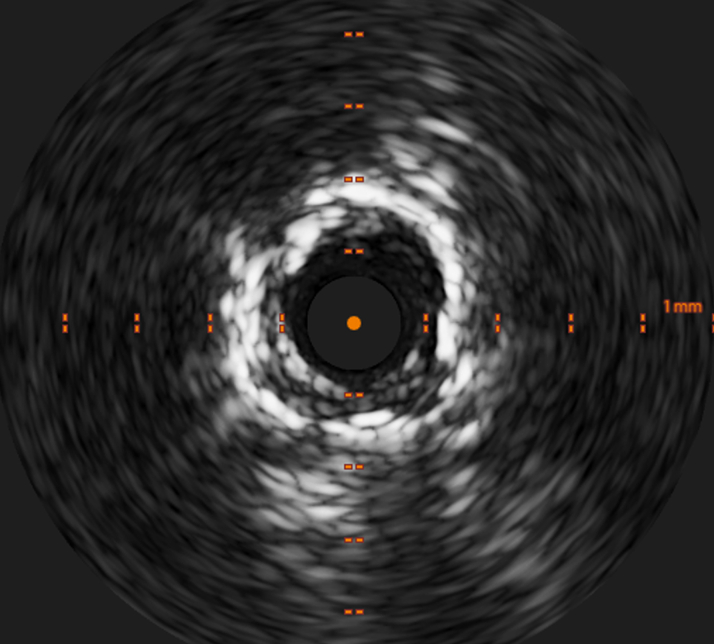
Intravascular Ultrasound Image Showing Underexpansion and Neointimal Hyperplasia Within the Stented Segment

The RCA was accessed using a Judkins Right 7-F guide catheter and Asahi Sion Blue wire. Predilation was performed with Euphora (Medtronic) 2.5 × 15-mm and 3 × 15-mm noncompliant balloons, followed by a Wolverine 4 × 10-mm coronary cutting balloon (Boston Scientific). Beta brachytherapy (23 Gy over 6 minutes, 7 seconds) was delivered to the restenotic segment, which resulted in satisfactory lesion preparation (Figure 1B). Intravascular ultrasound (IVUS) confirmed underexpansion and neointimal hyperplasia within the stented segment (Figure 2).

**Figure 3:**
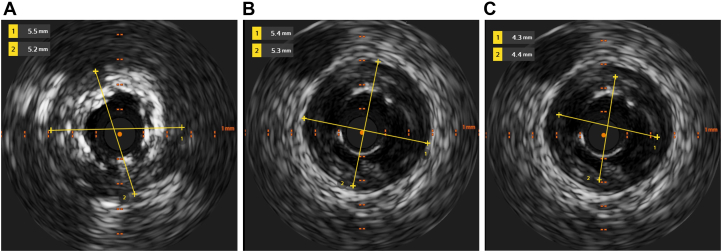
Intravascular Ultrasound Images (A) Proximal vessel diameter, (B) distal vessel diameter, and (C) lumen diameter.

Based on IVUS images of proximal and distal vessel and lumen diameters (Figure 3), we decided to further prepare the lesion with an Emerge 5 × 8-mm noncompliant balloon (Boston Scientific). However, post dilation, the patient developed acute chest discomfort. Repeat angiography revealed a stent fracture (Figure 1C) and Ellis type III perforation (Figure 1D). IVUS confirmed a type IV stent fracture—a complete transverse fracture with stent displacement and a visible gap (Figure 1E).

We performed prolonged balloon tamponade, which failed to achieve hemostasis. Consequently, a PK Papyrus 5 × 26-mm covered stent (Biotronik) was deployed at the perforation site, sealing the leak and restoring vessel integrity (Figure 1F). No further extravasation was seen. Transthoracic echocardiography revealed no pericardial effusion.

## Follow-Up

The patient remained stable and was discharged on dual antiplatelet therapy the next day. At 1-month follow-up, he remained symptom-free with no adverse cardiac events.

## Discussion

Stent fracture is a rare complication of percutaneous coronary intervention, reported in 1% to 8% of cases, associated with adverse cardiovascular events.^1^^,^^2^ The classification includes type IA (single-strut fracture), type IB (gap between 2 struts >2 times a 2.5-mm cell), type II (incomplete transverse, “V” gap), type III (complete transverse, no displacement), and type IV (complete transverse fracture with displacement).^2^ Type IV is severe due to its association with perforation, thrombosis, and restenosis.^2^

Several risk factors likely contributed to the stent fracture in this case. First, studies have shown that RCA stents, stainless stent, stent length >25 mm, hinge motion, overlapping, and multiple stents were independent predictors of stent fracture.^2^ Second, while brachytherapy is a frequent treatment choice for recurrent in-stent restenosis, radiation may alter the compliance of the vessel wall, contributing to increased stiffness and susceptibility to mechanical trauma.^3^^,^^4^ Third, this case was complicated by multiple overlapping stents requiring aggressive balloon expansion with a 5.0 balloon—all factors contributing to mechanical stress on stent struts. Finally, the RCA is especially susceptible to flexion, torsion, and shear stress during cardiac motion, increasing the risk of fracture and coronary perforation.^2^^,^^5^

In this case, the presence of extravasation on angiography and IVUS indicates an Ellis type III perforation (frank streaming of contrast through a >1-mm exit hole).^6^ Immediate goals are sealing the defect, maintaining coronary flow, and preventing cardiac tamponade.^6^ While the initial steps included balloon tamponade, it failed to control the perforation. Thus, the most appropriate strategy was the implantation of a covered stent.^6^ Urgent coronary artery bypass graft surgery remains a last-resort option, reserved for failed percutaneous interventions or when the anatomy is unsuitable for covered stents.

## Conclusions

This case highlights a rare but serious complication of percutaneous coronary intervention—type IV stent fracture leading to Ellis III coronary artery perforation. It underscores the risk posed by overlapping long stents, aggressive ballooning, and adjunctive therapies like brachytherapy. Prompt recognition of contrast extravasation, early use of IVUS, and rapid deployment of a covered stent prevented further deterioration and avoided surgical intervention.

## Funding Support and Author Disclosures

The authors have reported that they have no relationships relevant to the contents of this paper to disclose.

## References

[1] Alexopoulos D., Xanthopoulou I. (2011). Coronary stent fracture: how frequent it is? Does it matter?. Hellenic J Cardiol.

[2] Kan J., Ge Z., Zhang J.-J. (2016). Incidence and clinical outcomes of stent fractures on the basis of 6,555 patients and 16,482 drug-eluting stents from 4 centers. JACC Cardiovasc Interv.

[3] Waksman R., Cheneau E., Ajani A.E. (2003). Intracoronary radiation therapy improves the clinical and angiographic outcomes of diffuse in-stent restenotic lesions: results of the Washington Radiation for In-Stent Restenosis Trial for Long Lesions (Long WRIST) Studies. Circulation.

[4] Savage M.P., Fischman D.L. (2023). Resistant drug-eluting stent restenosis and resurrection of intracoronary brachytherapy: the lazarus of contemporary coronary intervention. Soc Cardiovas Angiogr Interv.

[5] Ramegowda R.T., Chikkaswamy S.B., Bharatha A. (2012). Circumferential stent fracture: novel detection and treatment with the use of StentBoost. Tex Heart Inst J.

[6] Ellis S.G., Ajluni S., Arnold A.Z. (1994). Increased coronary perforation in the new device era. Incidence, classification, management, and outcome. Circulation.

